# 

**Published:** 1853-08

**Authors:** 


### TABLE OF CONTENTS.

PAGE.

Acute Enterites. By Dr. W S Linn, 44
Armor Prof.—Prize Essay,...... 62-101
Aphonia by Iodine,............... 109
Association for the advancement of Science,..................... 125
Alum an emetic in poisoning with opium,......................... 149
Acknowledgement,................ 157
Arnold Prof.—Address to Graduates, 257
Amazon, Exploration of 263–298–325–357
Atkinson Dr.—Diseased Placenta,... 267
American Med. Association, 252–286–310
Antiperiodic properties of Humulus,.. 79
Aphonia relieved by Iodine,......... 109
Abscess Mammary, Iodine as preventative,........................ 147
Air Passages—Foreign body in. By G. R. Henry, M. D.,........... 203
Are the subjects of convulsions conscious ?...................... 239
Alcohol in Medicine,.............. 349
Bronchial glands, fatal disease of.... 120
Burns,........................... 289
Congestion of left lung. By Prof. M'Gugin,..................... 33
Croup, diptheritic. By M. Snider, M. D.,......................... 47
Creosote Inhalation, in phthisis,..... 76
Chloroform in obstruction of bowels,. 78
Chloride Soda, use of.............. 86
Chlorate of Potash in Cancrum Oris,. 87
College session—the Journal,... 124–380
Catamenia, early appearance of. By P. Van Patten, M. D.,......... 131
Cholera Epidemic. By J. Haines, M. D.,......................... 153
Chloroform, externally and internally, 175
" Deaths from............ 183
" Hooping Cough,......... 216
Clinical Lecture. By Prof. M'Gugin, 228
Chloroform, internal use of......... 242
Contributions to Pharmacy,......... 269
Common Salt in Intermittent Fever,. 293
Circulation of blood–new motive power 331
Causes of Sciatica,................ 337
Chloride of Sodium in Intermittent Fever,........................ 371
Chloroform, an Innocuous Anæsthetic 346
Dry Cupping. By A. H. Washington, M. D.,.................... 15

PAGE.

Dislocation of elbow. By Prof. Hughes 78
Dysentery, non-recurrence of........ 111
Dysentery, cold water in........... 82
Delirium relieved by Chloroform,.... 116
Dysentery. By W. S. Linn, M. D., 161
Dental Department,............... 220
Distribution of pulmonary vessels,... 334
Erysipelas, phlegmonous and gangrenous. By Prof. M'Gugin,..... 8
Erysipelas, treatment with Iron,..... 73
Ergot, uva ursi substitute for........ 75
Editorial,.................... 153–189
Ergot, causing retention placenta..... 171
Empiricism, Professional. By G. W. Hall, M. D.,................ 204
Erysipelas, analysis of............. 206
Education in Iowa,............... 221
Eagerness for practice,............ 247
Elements of professional character. By Prof. Arnold,............... 257
Epidemic Yellow Fever,............ 276
Excretions as guides to treatment in Rheumatism,................ 368
Fæcal obstruction, observations on... 143
Faculty, change in................ 156
Furunculus,...................... 336
Ford, Prof.—Absence,............. 348
Glossitis, a case of................ 109
Graduation of Students,........... 255
Hall on Spinal irritation,........... 13 ✗
Hughes on Dislocation elbow,....... 71
Hooping Cough, Nitric Acid in...... 85
Importance of Medical education. By Prof. Sanborn,............... 1
Insane of Iowa. By Prof. M'Gugin,. 65
Ipecac, External use of............ 86
Iowa Medical Department,.......... 91
Iodine, a preventive of Mammary abscess,........................ 147
Inunction in Scarlatina............ 216
Insane of Iowa,.................. 286
Infernal Practice................. 307
Indiana Medical Journal,......... 381
Jessamine, yellow................. 117
Journal,................ 223–256–380
Knowles, Prof.—on Med. Literature, 38
Letter from Prof. Hughes,........ 20–58
Linn on acute enteritis,............ 44
Letter to a young physician. By Prof Sanborn,.................... 97

PAGE.
Laryngeal and Pharyngeal diseases,.. 181
M'Gugin on Erysipelas,........... 8
Medical department of Iowa University,........................ 32
M'Gugin on congestion of left lung,.. 33
Monthly Memoranda,.. 54–88–121–153–189–217–249–349
Medical Miscellany, 64–96–128–168–224 288–352
M'Gugin, Prof.—Extract from Introductory,.................... 132
Mammary Inflammation,.......... 150
Medical obituary,.................. 159
" Students,.................. 220
Mott, Prof.—Quacks and Quack Nostrums,........................ 272
Needle Case,..................... 157
Our Journal,.................. 57–347
Obituary,........................ 126
Opium in anæmic states of brain,.... 138
One Certain Emmenagogue,........ 374
✗ Professional pebbles. By Prof. Sanborn,..................... 41–225
Phthisis, cod liver oil in........... 81
Puerperal convulsions, a case of...... 129
Physician's life,.................. 212
Pneumonia, treatment of........... 245
Placenta, disease of. By E. C. Atkinson, M. D.,.............. 267
Parisian Correspondence,.......... 281
Pharyngitis,...................... 287
Puerperal Fever. By P. S. Field, M. D.,...................... 287
Punctured fractures of Cranium. By Prof. Hughes,.............. 321
Physiology and Pathology of Pancreas 335
Quackery extraordinary,........... 191
Quinine, new method of administering 287
Retention of urine, ergot in......... 101
Rheumatism, the treatment of with Citric Acid,.................. 148

PAGE
Request,.......................... 159
Rheumatism, Lemon juice in........ 178
Re-vaccination. By G. Benedict, M D, 210
Raving Rhymes,.................. 222
Reflections on the life of medical men 345
Spinal irritation. By Dr. G. W. Hall,........................ 13
Salutatory,..................... 17
State Medical Society,............ 30
Stomatitis, Potash in............. 109
Sea Sickness, physiology of........ 80
Surgery, two cases of............. 113
Stomatitis Materni,.............. 84
Strychnia in nervous debility,....... 87
Sympathetic vomiting, chloroform in 151
Suum Cuique,................... 156
Surgery, cases in. By Prof. J. C. Hughes,....................... 168
Syphilis. By J. Grafton, M. D.,.... 197
Small Pox, Tincture Iodine in...... 214
Styptic Balsam,................. 243
Stimulus in Cholera, external...... 244
Suggestions to young physicians,.... 301
State Medical Society,........... 320
Starched Apparatus,.............. 331
Separation of the Symphysis Pubes,.. 378
Throat and lungs, diseases of....... 107
Tape worm, oil pumpkin seeds in.... 118
Turpentine, a uterine excitant...... 151
Tetanus. By Prof. Knowles,....... 193
Transfusion,.................... 232
Treatment of Rheumatism,......... 237
Typhus Fever,.................. 241
Traumatic Tetanus,.............. 342
Treatment of Scarlet Fever,........ 362
The College,.................... 380
Use of Chloroform,.............. 280
Vaccine Virus,.................. 287
Veratrum Viride. By A. Hard, M D 291
Correspondence with A. Hard, M. D. 353
Wound of carotid, external......... 229

☞ This Journal will be sent to those who do not, through their Post Master give notice of a desire for a discontinuance.

---

☞ A notice of the City Drug Stores, intended for this number, is unavoidably postponed for want of room.

---

### CONTENTS OF AUGUST NO.

Sanborn on Medical Education,.......... Page 1

McGugin on Erysipelas,.......... 8

Hall on Spinal Irritation,.......... 13

Washington on Dry Cupping,.......... 15

Editorial Salutatory,.......... 17

Dr. Hughes' Letter,.......... 20

Medical Department of Iowa University,.......... 27

Changes in Medical Faculty,.......... 29

State Medical Society.......... 30

